# Greater myofibrillar protein synthesis following weight-bearing activity in obese old compared with non-obese old and young individuals

**DOI:** 10.1007/s11357-023-00833-2

**Published:** 2023-06-17

**Authors:** Paul T. Morgan, Benoit Smeuninx, Ryan N. Marshall, Marie Korzepa, Jonathan I. Quinlan, Jamie S. McPhee, Leigh Breen

**Affiliations:** 1 School of Sport, Exercise and Rehabilitation Sciences, University of Birmingham, Edgbaston, Birmingham, B15 2TT UK; 2 Department of Sport and Exercise Sciences, Institute of Sport, Manchester Metropolitan University, 99 Oxford Road, Manchester, M1 7EL UK; 3 Monash Institute of Pharmacological Sciences, Monash University, Parkville, VIC Australia; 4 NIHR Birmingham Biomedical Research Centre, University Hospital Birmingham NHS Foundation Trust and University of Birmingham, Birmingham, UK; 5 MRC-Versus Arthritis Centre for Musculoskeletal Ageing Research, University of Birmingham, Birmingham, UK

**Keywords:** Fat mass, Intramuscular fat, Muscle mass, Muscle quality, Muscle protein synthesis, Obesity

## Abstract

The mechanisms through which obesity impacts age-related muscle mass regulation are unclear. In the present study, rates of integrated myofibrillar protein synthesis (iMyoPS) were measured over 48-h prior-to and following a 45-min treadmill walk in 10 older-obese (O-OB, body fat[%]: 33 ± 3%), 10 older-non-obese (O-NO, 20 ± 3%), and 15 younger-non-obese (Y-NO, 13 ± 5%) individuals. Surface electromyography was used to determine thigh muscle “activation”. Quadriceps cross-sectional area (CSA), volume, and intramuscular thigh fat fraction (ITFF) were measured by magnetic resonance imaging. Quadriceps maximal voluntary contraction (MVC) was measured by dynamometry. Quadriceps CSA and volume were greater (muscle volume, Y-NO: 1182 ± 232 cm^3^; O-NO: 869 ± 155 cm^3^; O-OB: 881 ± 212 cm^3^, *P* < 0.01) and ITFF significantly lower (*m.* vastus lateralis, Y-NO: 3.0 ± 1.0%; O-NO: 4.0 ± 0.9%; O-OB: 9.1 ± 2.6%, *P* ≤ 0.03) in Y-NO compared with O-NO and O-OB, with no difference between O-NO and O-OB in quadriceps CSA and volume. ITFF was significantly higher in O-OB compared with O-NO. Relative MVC was lower in O-OB compared with Y-NO and O-NO (Y-NO: 5.5 ± 1.6 n·m/kg^−1^; O-NO: 3.9 ± 1.0 n·m/kg^−1^; O-OB: 2.9 ± 1.1 n·m/kg^−1^, *P* < 0.0001). Thigh muscle “activation” during the treadmill walk was greater in O-OB compared with Y-NO and O-NO (Y-NO: 30.5 ± 13.5%; O-NO: 35.8 ± 19.7%; O-OB: 68.3 ± 32.3%, *P* < 0.01). Habitual iMyoPS did not differ between groups, whereas iMyoPS was significantly elevated over 48-h post-walk in O-OB (+ 38.6 ± 1.2%·day^−1^, *P* < 0.01) but not Y-NO or O-NO (+ 11.4 ± 1.1%·day^−1^ and + 17.1 ± 1.1%·day^−1^, respectively, both *P* > 0.271). Equivalent muscle mass in O-OB may be explained by the muscle anabolic response to weight-bearing activity, whereas the age-related decline in indices of muscle quality appears to be exacerbated in O-OB and warrants further exploration.

**Supplementary Information:**

The online version contains supplementary material available at 10.1007/s11357-023-00833-2.

## Introduction

The precipitous age-related loss of skeletal muscle mass, quality, and strength (“sarcopenia”) has well-documented health consequences for older adults that are associated with a significant societal and economic cost [16]. Alarmingly, the number of individuals presenting with this musculoskeletal disease is increasing exponentially with population ageing [16]. Increasing adiposity also occurs with advancing age [31], resulting in an unfavourable ratio of fat to muscle that is associated with poor overall health, reduced functional capacity, and lower quality of life alongside heightened risk of metabolic syndrome, type II diabetes, and cancer [29]. Importantly, the health complications arising from sarcopenia and obesity are thought to be multiplicative [2]. Indeed, sarcopenia progression is accelerated in obese older individuals [12, 33, 48], while sarcopenic obesity (the confluence of sarcopenia and obesity) heightens the risk of many chronic diseases and all-cause mortality [37].

Sarcopenia is largely underscored by a blunted muscle protein synthesis (MPS) response to amino acid nutrition, termed “anabolic resistance” [34], which is exacerbated by the presence of obesity [5, 50, 61]. Despite this, measures of absolute lean mass have been shown to be equivalent or greater in obese compared with non-obese older adults (e.g. [61]. Although relative lean tissue mass may, in some cases, remain lower in obese individuals due to their larger body mass [52], these data suggest that obesity in older age may offer some partial protection for absolute levels of lean mass. However, the mechanisms that underpin this phenomenon remain unclear. MPS is highly responsive to contractile loading (e.g. resistance exercise) and exercise/physical activity is a potent stimulus for muscle remodelling and maintenance in older adults [1]. In obese older adults, the requirement to move more inert mass during weight-bearing activities (e.g. walking as part of activities of daily living) could induce sufficient loading forces to stimulate MPS and offset potential age-related muscle mass loss caused by anabolic resistance to amino acids [48, 55], which may counteract amino acid-induced anabolic resistance to explain equivalent or greater absolute muscle mass in obese vs. non-obese older individuals.

While the loss of muscle mass is significantly related to loss of strength and function [73], the age-related decline in muscle strength and, therefore, quality (strength/function relative to muscle mass) may occur more rapidly than the loss of muscle mass [24, 42, 44]. The deterioration in muscle quality may be more apparent in obese older individuals, potentially through lipid-induced alterations to muscle contractile properties [63] and a requirement to move greater body mass [48, 68], although not all studies support this notion [33]. Importantly, muscle quality is strongly associated with numerous health outcomes [36] and strength/function has been consistently associated with lower all-cause mortality risk in later life (e.g. [58]. While the presence of obesity in older age may be associated with equivalent or greater absolute muscle mass compared with non-obese older individuals, muscle quality may be worsened. To date, the “trade-off” between muscle mass and indices of muscle quality in obese older individuals has not been fully explored and requires clarification. Studies to date have also largely been limited to dual-energy x-ray absorptiometry measures lean tissue mass rather than muscle mass, per se (e.g. [61],Tomlinson, Erskine, Winwood, et al. 2014a).

The primary purpose of this study was to investigate rates of integrated myofibrillar protein synthesis (iMyoPS) prior-to and following a weight-bearing walking task in older obese (O-OB) compared with older non-obese (O-NO) and younger non-obese (Y-NO) individuals. Quadriceps muscle mass, intramuscular fat, maximal strength, and voluntary activation were measured as indices of muscle quality in O-OB, Y-NO, and O-NO. Our primary hypothesis was that the iMyoPS response to weight-bearing activity would be greater in O-OB compared with O-NO and Y-NO, with no differences between O-NO and Y-NO. In addition, we hypothesised that the iMyoPS response to weight-bearing activity would be significantly greater compared with habitual free-living iMyoPS in O-OB but not O-NO and Y-NO. Lastly, we posited that quadriceps muscle mass would be equivalent or greater, intramuscular fat greater, and indices of muscle quality poorer in O-NO compared with Y-NO and exacerbated in O-OB.

## Methods

### Participants

Fifteen young non-obese (Y-NO; 23 ± 4 years, BMI; 24 ± 3 kg·m^−2^, body fat: 13 ± 5%), 10 older non-obese (O-NO; 69 ± 5 years, BMI; 25 ± 2 kg·m^−2^, body fat: 20 ± 3%), and 10 older obese (O-OB; 73 ± 5 years, BMI; 31 ± 3 kg·m^−2^, body fat: 33 ± 3%, Table 1) consenting male adults participated in the present study. Participants were recruited via word of mouth and public engagement groups. The inclusion of Y-NO was intended as a young healthy reference to investigate the effect of isolated and combined ageing and obesity influenced the muscle anabolic response to weight-bearing activity. We did not expect that iMyoPS would be stimulated above habitual levels following weight-bearing activity in Y-NO. Participants completed a general health questionnaire and were excluded if they had uncontrolled hypertension, neuromuscular/cardiovascular/metabolic disease, or if they were smokers, currently losing weight and/or consuming regular non-steroidal anti-inflammatory or blood-thinning medication. After being informed of the experimental procedures and associated risks, all participants completed a medical health screening which incorporated assessments of blood pressure and a 12-lead electrocardiogram (ECG) to ensure they could safely perform strenuous exercise. In addition, all participants were required to demonstrate satisfactory physical function, as determined via a score of ≥ 9 according to the Short Physical Performance Battery (SPPB). No participants were excluded as a result of low/poor physical function. The COVID-19 global pandemic impacted study recruitment and the duration of the trial. The study was approved by the NHS Research Ethics Edgbaston Committee (Ref: 18/WM/0234) and conformed to the requirements of Research Governance at the University of Birmingham, as the study sponsor (Ref: RG_18-090) and to the principles of the World Medical Association Declaration of Helsinki.

**Table 1:** Anthropometric, SPPB, body composition, and metabolic health data

	Y-NO	O-NO	O-OB
Age (years)	23 ± 4	69 ± 5 *	73 ± 5 ^*^
Body mass (kg)	74.4 ± 10.2	77.6 ± 5.2	91.0 ± 8.0 * ^#^
Systolic blood pressure (mmHg)	120 ± 6	128 ± 9 *	136 ± 16 *
Diastolic blood pressure (mmHg)	71 ± 8	78 ± 7	77 ± 10
BMI (kg·m^−2^)	23.8 ± 2.6	25.2 ± 1.7	31.2 ± 2.7 * ^#^
SPPB (AU)	-	11 ± 1	11 ± 1
Habitual walking speed (m/s)	-	0.93 ± 0.13	1.02 ± 0.21
Fat mass (% of body mass)	13.0 ± 5.2	20.0 ± 2.8 *	33.0 ± 2.9 * ^#^
Fat mass (kg)	9.7 ± 4.2	15.5 ± 2.0 *	30.0 ± 2.9 * ^#^
Leg fat mass (kg)	3.9 ± 2.1	4.2 ± 0.9	7.7 ± 1.7 * ^#^
Whole-body FFM (kg)	62.7 ± 8.9	60.1 ± 6.6	61.2 ± 3.8
Whole-body FFM (% of body mass)	84.3 ± 6.5	77.4 ± 7.5 *	67.3 ± 4.0 * ^#^
Whole-body SMM (kg)	29.7 ± 3.5	26.3 ± 2.7	27.0 ± 1.9
Whole-body SMM (% of body mass)	39.9 ± 3.7	33.9 ± 3.1 *	29.7 ± 2.4 * ^#^
HOMA-IR	1.6 ± 0.3	1.5 ± 0.2	3.6 ± 1.9 * ^#^
Fasting plasma glucose (mmol/L)	4.6 ± 0.3	4.8 ± 0.5	5.5 ± 1.1 *
Fasting serum insulin (pmol/L)	54.4 ± 10.0	48.3 ± 10.9	92.6 ± 56.3 * ^#^
Plasma HbA1C (mmol/mol)	35 ± 2	36 ± 2	38 ± 5
Plasma IL-6 (pg/mL)	0.40 ± 0.30	0.94 ± 0.43 *	1.39 ± 0.66 * ^#^
Plasma CRP (mg/L)	0.35 ± 0.15	0.68 ± 0.24 *	1.58 ± 0.67 * ^#^

Data presented as mean ± standard deviation. SMM (%) presented relative to total body weight. * Significantly different from Y-NO, ^#^ significantly different from O-NO

Abbreviations: *Y-NO*, young non-obese; *O-NO*, older-non-obese; *O-OB*, older obese; *HOMA-IR*, homeostasis model assessment of insulin resistance; *FFM*, fat free mass; *SMM*, skeletal muscle mass; *SPPB*, short physical performance battery; *AU*, arbitrary units; *RMR*, resting metabolic rate

### Experimental design

**Fig. 1:**
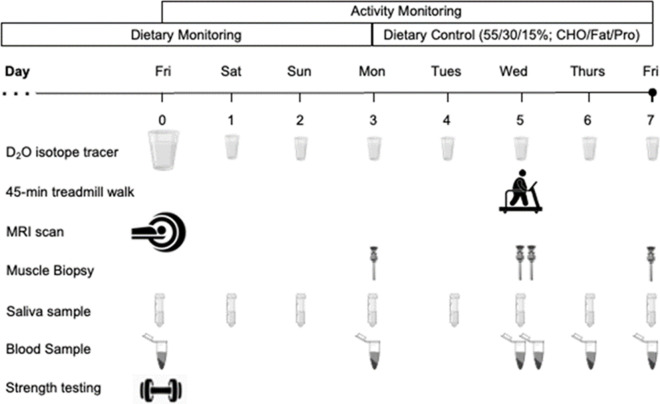
Study schematic and overview of experiential procedures during the muscle protein synthesis assessment period. D_2_O, deuterium oxide (deuterated water); MRI, magnetic resonance imaging; CHO, carbohydrate; Pro, protein. Drawn using BioRender©

Y-NO, O-NO, and O-OB were recruited to investigate the role of obesity on rates of integrated myofibrillar protein synthesis (iMyoPS) and indices of muscle mass, quality, function, and morphology. Participants reported to the National Institute for Health Research/Wellcome Trust Clinical Research Facility (CRF) of the Queen Elizabeth Hospital (Birmingham, UK), the Centre for Human Brain Health ([CHBH] University of Birmingham, UK), and/or the Sport, Exercise and Rehabilitation Sciences Laboratory (University of Birmingham, UK) on 7 occasions over a ~ 3-week period (Fig. 1). For visits 3–7, described below, participants reported to the lab in an overnight fasted state at ~ 08:00 h (± 2 h). In addition, participants were asked to refrain from any strenuous physical activity and alcohol consumption throughout the iMyoPS assessment period (Fig. 1).

### Experimental protocol

#### Visit 1: enrolment and eligibility

Following a telephone screening, participants reported to the CRF. On arrival, participants completed a general health questionnaire verified by a medical professional. Anthropometrics and compartmental body composition (including whole-body skeletal muscle mass) were assessed, the latter via bioelectrical impedance analysis using a segmental body composition analyser (BC-418, Tanita Europe BV, Amsterdam, The Netherlands) incorporating assessments of segmental fat mass, body fat percentage, fat-free mass, and predicted skeletal muscle mass to verify eligibility and to stratify participants into respective groups. Finally, an SPPB was conducted to assess participants’ function. The SPPB is a series of measures that combines the results of gait speed, chair stand, and balance tests, and typically used as a predictive tool for possible disability in older people.

#### Visit 2: progressive “walk-to-run” test

Three-to-seven days after visit 1, participants returned to the laboratory to complete a progressive “walk-to-run” exercise test with 2-min stages (modified Bruce protocol, Supplemental Table 1) until volitional exhaustion for determination of maximal oxygen uptake (i.e. V̇O_2peak_). Each test was supervised by the research lead, a medical physician and research nurses. The test was terminated voluntarily by the participant when they could no longer continue the prescribed speed and incline despite strong verbal encouragement. Pulmonary gas exchange and ventilation were measured throughout using a metabolic cart connected to a mouthpiece assembly (Moxus Metabolic Cart; AEI Technologies, Pittsburgh, PA, USA). The final speed and incline were recorded at the end of the test and used to estimate the work rate for the 45-min treadmill task in visit 5, described below. During the test, participants wore shorts fitted with textile electromyography (EMG) electrodes to quantify the electric potential of quadriceps and whole-thigh muscle and referred to as “quadriceps muscle EMG activity” and “thigh muscle EMG activity”, respectively, hereafter. Briefly, surface EMG was recorded from the right and left thigh (quadriceps, hamstrings, glutes) using textile shorts made of polyamide (71%) and elastane (29%) with bipolar electrodes integrated into the fabric (Mbody, MyonTec Ltd, Kuopio, Finland). A range of sizes were used to ensure close proximity of the electrodes to the skin and to maximise the sensitivity of the EMG signal. The skin area underneath each EMG electrode was covered with conductive gel to optimise conductivity between the skin and electrodes. Prior to being discharged, participants were provided with a wrist-worn accelerometer with a triaxial sensor (GENEActiv; ActivInsights, Huntingdon, UK) and a wrist-worn pedometer (ID115HR LETSCOM, Hong Kong) for 7–10 consecutive days to measure physical activity levels, and a diary to record food intake.

#### Visit 3: day 0

Three-to-seven days after visit 2, participants returned to the laboratory following an overnight fast (see Fig. 1: day 0). An initial blood sample was obtained from an antecubital vein to determine markers of inflammation and metabolic health (e.g. HOMA-IR, fasting plasma glucose, serum insulin, plasma HbA1C, plasma IL-6, plasma CRP), as previously described [61]. Thereafter, once a saliva sample (~ 5 mL) was obtained, participants consumed a bolus (2.4 mL·kg of lean body mass^−1^) of an oral stable isotope tracer (deuterated or “heavy” water [D_2_O], 70 atom%; Cambridge Isotope Laboratories, Inc, Tewksbury, MA) over ~ 60 min to rapidly increase the body water enrichment of deuterium (^2^H) to ∼ 0.2% atom percent excess (APE) for detection in muscle and saliva for the measurement of iMyoPS via mass spectrometry, as previously described [43] (see the “Muscle protein synthesis” section). Participants were then transported to the CHBH to measure quadriceps-specific and total thigh muscle cross-sectional area (CSA) and muscle volume, intramuscular thigh fat fraction (ITFF), and patella tendon moment arm length via a magnetic resonance imaging (MRI) scanner (Siemens MAGNETOM Prisma 3 T MRI system, Siemens, Munich, Germany). Specifically, MRI was used to collect transverse plane Sects. (3.00 mm slice thickness, 3.97 ms repetition time [TR], 9° flip angle, 1.29 ms slice echo time [TE]) from the right leg tibial tubercle through to the anterior–inferior iliac spine, with participants in a supine position on the scanner bed for assessment of thigh CSA, thigh muscle volume, and ITFF. Participants entered the magnet feet first, with multiple 16-channel body coils positioned over their thighs and patella. A 3-plane localiser scan was used to prepare the fast-spin-echo scans using the DIXON sequence. The total number of 3.00 mm images acquired was ~ 192 but varied depending on the length of the participants’ thighs. Immediately following transverse plane imaging of the thigh, while lying in the same position, sagittal plane sections of the right knee were collected (slice thickness: 3.00 mm, TR: 500 ms, TE: 10 ms, flip angle: 124°) to measure patella tendon moment arm length for the assessment of in-situ muscle-specific force. Finally, participants returned to the laboratory for an assessment of isometric leg strength and voluntary activation (VA) of the right quadriceps, determined via isokinetic dynamometry (Biodex System 3, Shirley, NY, USA), as previously described [49]. Briefly, participants completed 3 × 3 s MVC of the knee extensors with each contraction separated by a 60 s passive recovery period. During MVCs, single peripheral nerve stimulation pulses (100 µs pulses at 400 V) were triggered to occur as soon as a peak torque was achieved to provide a “superimposed twitch” (sTw). The stimuli were also delivered 2 s after the cessation of the MVC to provide a potentiated “resting twitch” (pTw). Electrical stimulation was applied by a constant current stimulator (Digitimer Stimulator DS7, Digitimer, UK). Prior to discharge, participants were provided with daily D_2_O “top-ups” (0.3 mL·kg of lean body mass^−1^) for the duration of the study to consume on waking for the next 7 consecutive days to ensure maintenance of a “steady state” of ∼ 0.2% APE deuterium enrichment of the body water pool throughout study involvement. Participants were also supplied with sterile containers to provide daily sample prior to consumption of their daily dose of D_2_O for later analysis of ^2^H body water enrichment.

#### Visits 4, 6, and 7: percutaneous skeletal muscle biopsy

Three days after visit 3, participants returned to the laboratory having provided a saliva sample and consumed the daily dose of D_2_O. Saliva samples were temporarily stored in the participant home fridge (3–5 °C) and returned to the investigator at each experimental visit, where they were subsequently centrifuged (11,000 g for 10 min at 4 °C) to separate salivary debris before storage at – 80 °C until later analysis. Thereafter, an initial skeletal muscle biopsy from the vastus lateralis and venous blood samples were obtained, as previously described [61]. Prior to departure, participants were provided with a “weight maintenance” food parcel (∼ 50% carbohydrate, ∼ 30% fat, and ∼ 20% protein) for the subsequent 4 days to standardise dietary conditions between groups during the period of iMyoPS assessment (Fig. 1, Supplemental Table 2). Energy requirements were calculated using the Harris-Benedict equation [27] multiplied by an activity factor which best reflected individual habitual activity patterns (range 1.2–1.9, but generally 1.35 for all groups). Participants could choose from a selection of food options and consisted of breakfast, lunch, dinner, and snacks to ensure regular intakes of protein throughout the day. Fruit juices were also provided and water permitted ad libitum. Participants then reported to the CRF 48 h later to complete a 45-min treadmill walk (described below). Visits 6 and 7 were completed 24 h and 48 h after visit 5, respectively, and replicated the experimental procedures detailed in visit 4.

#### Visit 5: 45-min treadmill walk

To determine the impacts of a weight-bearing task on skeletal muscle properties, participants completed a 45-min treadmill walk at ~ 55% of V̇O_2peak_. This intervention was selected to mimic the demands of a brisk walking activity completed as part of typical activities of daily living. A similar intensity-duration treadmill walk task was previously shown to modestly increase MPS above basal rates in non-obese older adults [65], which we expected might increase more robustly when performed by O-OB. Briefly, the treadmill speed and gradient were adjusted to ensure a constant intensity throughout the 45 min, with participants wearing the mouthpiece assembly, as described in visit 2, at intermittent periods to monitor oxygen consumption. During treadmill walking, participants wore shorts fitted with textile EMG electrodes to quantify quadriceps muscle and whole-thigh muscle activation patterns (MShorts-AllSport, MyonTec Ltd, Finland). The EMG shorts were calibrated against maximal EMG achieved during visit 2. Prior to and immediately post treadmill walk, skeletal muscle biopsies from the vastus lateralis and venous blood samples were obtained. Prior to discharge, participants consumed a post-exercise beverage containing ~ 23 g of whey isolate protein (Myprotein, Northwich, UK) to augment exercise-induced MPS similarly in the initial hours of recovery.

### Muscle protein synthesis

#### Integrated myofibrillar fractional synthetic rate and intramuscular signalling

Habitual (visits 4 and 5) and post-walk (visits 5–7) fractional protein synthetic rates (FSR) were determined by measurement of deuterium enrichment in the myofibrillar protein pool via gas chromatography-pyrolysis-isotope ratio mass spectroscopy. On extraction of all muscle tissue, biopsy samples were quickly rinsed in ice-cold saline and blotted to remove any visible blood, fat, and connective tissue before being immediately snap frozen in liquid nitrogen and stored at – 80 °C until analysis. The reader is directed to our previous work which provides more detailed description of our isotope tracer protocol and experimental approaches to assess body water enrichment and myofibrillar protein enrichment (i.e. assessment of myofibrillar bound ^2^H-alanine) [43]. Alanine was used as the amino acid for analysis due to its abundance, multiple sites of incorporation (e.g. muscle, plasma, saliva), and close representation of the total amino acid pool. Therefore, FSR was calculated from the enrichment of alanine, as calculated from body water enrichment, and the enrichment of alanine in the myofibrillar proteins and extrapolated to the entire amino acid pool. Briefly, ~ 30 mg of tissue was rapidly homogenised prior to separation of the myofibrillar-enriched portion from mitochondrial, collagen, and sarcoplasmic muscle fractions. Once the myofibrillar-enriched fraction had been washed and purified, protein‐bound amino acids were released using acid hydrolysis and left to hydrolyse at 110 °C overnight, before being eluted from resin with ammonium hydroxide (NH_4_OH) on cation-exchange columns. Thereafter, samples were dried and reconstituted in hydrochloric acid (HCl) before analysis by gas chromatography-pyrolysis-isotope ratio mass spectroscopy (Metabolic Solutions, Nashua, NH, USA). Anabolic (i.e. mTOR-mediated signalling) intramuscular signalling markers were determined for total (4E-BP1, AMPK, Akt, rpS6, IRS-1, TSC2) and phosphorylation (4E-BP1^Thr37/46^, AMPK^Thr72^, Akt^Serine 473^, rpS6^Ser235/236^, IRS-1^Ser636/639^, TSC2^Thr1462^) protein content by western blot analysis on the sarcoplasmic protein fraction obtained during myofibrillar protein isolation, as previously described (e.g. [43, 61] (see supplementary methods for primary antibodies for immunoblotting).

#### Calculation of fractional synthetic rate

iMyoPS was calculated using the standard precursor-product method, from the incorporation of deuterium‐labelled alanine into protein, using the enrichment of body water (corrected for the mean number of deuterium moieties incorporated per alanine and the dilution from the total number of hydrogens in the derivative) as the surrogate precursor labelling between subsequent biopsies. FSR was calculated as follows:

$$\mathrm{FSR}(\mathrm{\%}/day) = [(\mathrm{APEAla})]/[(\mathrm{APEP}) \times t] \times 100$$

where APE_Ala_ is deuterium enrichment of protein‐bound alanine, APE_P_ is mean precursor enrichment over time, and *t* is the time between biopsies.

#### Data analysis

V̇O_2peak_ during the progressive “walk-to-run” test was determined as the highest 30-s mean value throughout. The intensity (speed/incline) which corresponded closest to 55% of V̇O_2peak_ was used as an estimate for the visit 5 walking task. For assessment of muscle activity (visits 2 and 5), EMG signals were pre-amplified (1000 Hz) and then band-pass filtered (2nd order) with cut-off frequencies of ~ 40–300 Hz. EMG data were recorded continuously and digitised synchronously with 12-bit resolution via an A/D converter. The 1000 Hz signal was first rectified and then averaged over non-overlapping 100 ms intervals, similar to the root mean square method. The averaged data was stored in the memory of the MCell at 25 Hz, from which the data was extracted and analysed using customised software (Muscle Monitor, MyonTec Ltd, Kuopio, Finland). All EMG data was visually assessed and corrected for artefacts. The maximal EMG signal for visit 2 was determined as the highest 30 s mean value. For visit 5, to obtain an average rectified value of EMG, the average EMG signal was taken from the onset of the 45-min exercise task until task end and presented relative to the maximal EMG achieved during the visit 2. Accelerometers, for physical activity characterisation, were initialised to sample data at a 10-Hz frequency. Data were converted into 60-s epochs and analysed using the GENEActiv software (version 2.2; ActivInsights, Huntingdon, UK). Activities were split into four categories based on metabolic equivalent (MET) values: (1) sedentary activity (< 1.50 METs), (2) light activity (1.50 to 3.99 METs), (3) moderate activity (4.00 to 6.99 METs), and (4) vigorous activity (> 7 METs). Commercially available software (ITK-SNAP version 4.0, PA, USA) was used to estimate the anatomical CSA of each of the four heads of the quadriceps muscles and total thigh muscle from transverse plane images at 6.00 mm intervals (i.e. every other slice) from the distal to the proximal ends of the quadriceps by manual segmentation. The top 30% (from the greater trochanter working distally) and bottom 20% (from the distal end of the femur working proximally) were excluded so that only the middle 50% region of the assessed area was used for manual segmentation. These CSA were summed and multiplied by the distance between slices to estimate quadriceps-specific and total thigh muscle volume. In addition, peak CSA and CSA at 50% (of femur length) were calculated. All analyses were conducted by the same investigator (PM), and every 10 slices were blind checked for quality control (JQ), with an interrater agreement of 0.97. For quadriceps-specific and total thigh intramuscular lipid quantification (i.e. ITFF), images acquired with a 2-point Dixon MRI sequence were analysed using Medical Image Processing, Analysis and Visualization ([MIPAV] National Institutes of Health, Bethesda, MD, USA) software to isolate regions of interest (ROI). The Dixon method is an MRI sequence based on chemical shift and designed to achieve uniform fat suppression. The Dixon technique exploits the fact that water and fat molecules were process at different rates (Dixon et al. 1984). A 2-point Dixon acquisition exploits the difference between resonance frequencies of hydrogen nuclei in water and fat molecules to produce four sets of images: water only, fat only, in phase, and out of phase. Calculation of fat fraction (%) was carried out blinded using the following formula:

1 $$\mathrm{Fat fraction}\left(\mathbf{\%}\right)= 100 \times \left( \frac{Fat mean intensity}{\mathrm{Water mean intensity }+\mathrm{ Fat mean intensity}}\right)$$

Briefly, ROIs were manually drawn around each respective muscle prior to analysis of signal intensity at the same location for water-only and fat-only captured images. ROIs were drawn in three consecutive slices at 50% limb length in the same manner, with the three values averaged for each participant. Due to issues pertaining to contraindications associated with MRI scans and the impact of COVID on in-person research activity, MRI outputs consisted of 29 datasets distributed within the groups as follows: 15/15 (Y-NO), 7/10 (O-NO), and 7/10 (O-OB). For assessment of quadriceps muscle force, for each 3 s contraction, maximal voluntary force was determined as the mean value over a 1 s period, which approximated the plateau level of the highest torque. The resting twitch torque was calculated as the peak torque achieved following the single pulse delivered 1 s post-MVC. The superimposed twitch torque was calculated as the increment in torque immediately following the pulse during MVCs. Voluntary activation (VA) was calculated using the interpolated twitch method. Specifically, the increment in torque (i.e. superimposed twitch) evoked during the MVCs was expressed as a fraction of the amplitude of the resting potentiated twitch produced with the same stimuli in the relaxed muscle post-MVC. The level of voluntary drive was then quantified as a percentage as previously described [47]. Finally, dietary intake was analysed via dietary records using DietPlan 7 software (Forestfield Ltd, West Sussex, UK).

#### Statistical analysis

Differences in baseline group characteristics and quadriceps muscle volume characteristics were assessed via one-way analysis of variance (ANOVA). Differences in integrated myofibrillar protein synthesis (iMyoPS) and intramuscular signalling were assessed using a mixed model ANOVA with one within (two levels—48-h habitual and 48-h exercise induced) and one between factor (three levels; group [Y-NO, O-NO, O-OB]). Where the ANOVA revealed a significant effect, post-hoc analysis was conducted using a Holm-Bonferroni correction to isolate specific differences. For calculation of effect size, partial eta squared (*η*^2^) was used for omnibus tests, where *η*^2^ = 0.01, 0.06, and 0.14 indicate small, medium, and large effects, respectively. Cohen’s *d* was used to calculate the effect size for post-hoc comparisons, where *d* = 0.2, 0.5, and 0.8 indicate small, medium, and large effects, respectively. Where sphericity was violated, a Greenhouse–Geisser correction factor was used. Normal distribution was assessed using the Shapiro–Wilk test. Where appropriate, non-normally distributed variables were logarithmically transformed. All other data were analysed using a one-way ANOVA. For all the tests, results were considered statistically significant when *P* < 0.05. Data are presented as mean ± standard deviation or standard error of the mean, unless otherwise indicated. All statistical analyses were conducted using IBM SPSS Statistics version 28.

## Results

### Physical activity, diet, and blood characteristics

**Fig. 2:**
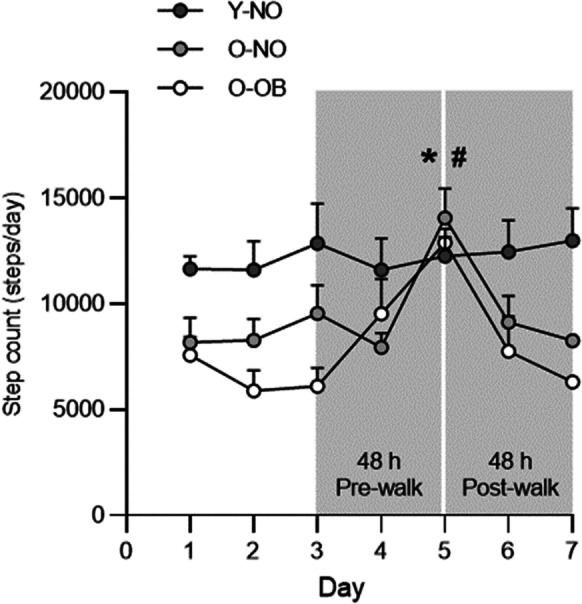
Step count during the 7-day MPS assessment period for young non-obese (Y-NO; dark grey circles), old non-obese (O-NO; light grey circles), and old obese (O-OB; clear circles) adults. The 48-h habitual (i.e., pre-walk) and 48-h exercise (i.e. post-walk) phases are shaded in grey. Average daily step count was significantly higher in Y-NO compared with O-NO and O-OB. Step count on day 5 was significantly elevated, above habitual levels, in O-NO and O-OB but not Y-NO. Significance was set at *P* < 0.05. * Significantly different from O-NO; # significantly different from O-OB. Values are presented as means ± SD

Anthropometrics, SPPB score, body composition, and metabolic health data for Y-NO, O-NO, and O-OB can be seen in Table 1. Body mass, BMI, whole-body fat mass, and leg fat mass were significantly higher in O-OB compared with O-NO and Y-NO (all *P* < 0.001), with no differences in body mass (*P* = 0.346, *d* = 0.41) and BMI (*P* = 0.136, *d* = 0.65) between Y-NO and O-NO (Table 1). Whole-body fat free mass and skeletal muscle mass were lower in O-OB and O-NO compared with Y-NO (all *P* < 0.001, *d* > 0.98) and were lower in O-OB compared with O-NO (*P* < 0.01; *d* > 1.52, Table 1). Plasma IL-6 and CRP were significantly elevated in O-NO (both *P* < 0.001, *d* > 1.40) and O-OB (both *P* < 0.001, *d* > 1.93) compared with Y-NO and greater in O-OB compared with O-NO (both *P* < 0.01, *d* = 0.81). HOMA-IR, fasting plasma glucose, serum insulin, and plasma HbA1C were significantly elevated in O-OB compared with Y-NO (Table 1, all *P* < 0.028, *η*^2^ > 0.26), but did not differ between O-NO and Y-NO. All participants achieved a score of ≥ 9 in the SPPB (Table 1), demonstrating a good level of physical function. Habitual physical activity characteristics and self-reported dietary intake are presented in Table 2. Average daily step count was significantly higher in Y-NO (*P* < 0.01, *η*^2^ = 0.33) compared with O-NO (*P* = 0.035, *d* = 0.92) and O-OB (*P* < 0.001, *d* = 1.70). Step count on the day of the treadmill walk (visit 5) was significantly elevated above all other days in O-NO and O-OB (*P* < 0.001) but not Y-NO (*P* = 0.986, Fig. 2).

**Table 2:** Habitual activity and self-reported diet characteristics

	Y-NO	O-NO	O-OB
Daily step count	12,261 ± 3026	9595 ± 2754 *	8571 ± 1363 *
Sedentary activity (%)	64.5 ± 4.4	70.5 ± 4.1 *	73.7 ± 7.6 *
Light activity (%)	12.5 ± 2.4	16.6 ± 5.9 *	13.2 ± 4.8
Moderate activity (%)	20.6 ± 3.4	12.6 ± 3.6 *	13.1 ± 4.0 *
Vigorous activity (%)	2.5 ± 1.2	0.3 ± 0.5 *	0.1 ± 0.1 *
Daily energy intake (kcal)	2739 ± 661	2179 ± 192 *	2113 ± 584 *
Daily protein (g⋅ kg^−1^)	1.84 ± 0.59	1.33 ± 0.39 *	0.94 ± 0.25 * ^#^
Daily CHO (g⋅ kg^−1^)	3.41 ± 1.04	2.98 ± 0.35 *	2.21 ± 0.66 *
Daily fat (g⋅ kg^−1^)	1.66 ± 0.67	1.12 ± 0.33 *	0.99 ± 0.30 *
Daily alcohol (g⋅ kg^−1^)	0.12 ± 0.06	0.15 ± 0.08	0.20 ± 0.26
Daily fiber (g⋅ kg^−1^)	0.40 ± 0.12	0.43 ± 0.08	0.22 ± 0.06 * ^#^

Data presented as mean ± standard deviation. * Significantly different from Y-NO, ^#^ significantly different from O-NO

Abbreviations: *Y-NO*, young non-obese; *O-NO*, older-non-obese; *O-OB*, older obese; *CHO*, carbohydrate

### Treadmill performance

V̇O_2peak_, as assessed during visit 2, was significantly higher in Y-NO (4.1 ± 0.6 L·min^−1^, 54.7 ± 7.2 mL·kg^−1^·min^−1^, *P* < 0.001, *η*^2^ = 0.81), with no differences between O-NO and O-OB (OL: 2.4 ± 0.5 L·min^−1^, 31.3 ± 7.0 mL·kg^−1^·min^−1^, O-OB: 2.5 ± 0.6 L·min^−1^, 26.7 ± 4.2 mL·kg^−1^·min^−1^, *P* = 0.128, *d* = 0.78). The average peak speed and incline achieved during the incremental test was 8.2 ± 0.5 km·h^−1^ and 18.3 ± 1.1%, 6.0 ± 0.7 km·h^−1^ and 14.0 ± 1.3%, and 4.7 ± 0.8 km·h^−1^ and 12.6 ± 1.0%, for Y-NO, O-NO, and O-OB, respectively. This corresponded to an average speed and incline of 4.6 ± 0.7 km·h^−1^ and 12.2 ± 1.6%, 3.8 ± 0.2 km·h^−1^ and 10.1 ± 1.1%, and 3.2 ± 0.5 km·h^−1^ and 6.6 ± 1.9% during the 45-min treadmill walk for Y-NO, O-NO, and O-OB, respectively.

### Quadriceps tissue composition

**Fig. 3:**
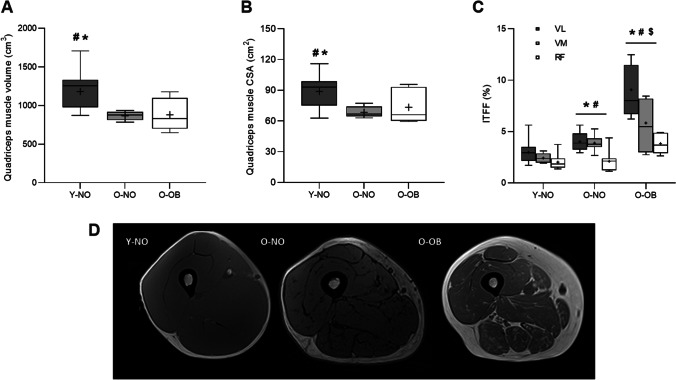
Quadriceps muscle volume (**A**) and peak quadriceps muscle cross sectional area (CSA, **B**) for young non-obese (Y-NO, dark grey bars), old non-obese (O-NO, light grey bars), and old obese (O-OB, white bars), respectively. Quadriceps muscle intramuscular thigh fat fraction (ITFF) for Y-NO, O-NO, and O-OB can be seen in **C**, respectively, for vastus lateralis (VL, dark grey boxes), vastus medialis (light grey boxes), and rectus femoris (white boxes). Values are presented as the median (central horizontal line), 25th and 75th percentiles (box), minimum and maximum values (vertical lines), and mean (cross). Representative CSA images at 50% of thigh length can be seen in **D** for Y-NO, O-NO, and O-OB, respectively. The young non-obese individual exhibits a larger quantity of skeletal muscle mass, whereas high infiltration by adipose tissue is observed in the older obese individual. Significance was set at *P* < 0.05. For **A** and **B**, * Significantly different from O-NO, # significantly different from O-OB. For **C**, * VL significantly different from Y-NO, ^#^ VM significantly different from Y-NO, ^$^ RF significantly different from Y-NO

There was a significant effect of group on quadriceps muscle volume (Y-NO: 1182 ± 232 cm^3^; O-NO: 869 ± 155 cm^3^; O-OB: 881 ± 212 cm^3^, *P* < 0.01, *η*^2^ = 0.41) and peak CSA (Y-NO: 89.0 ± 14.4 cm^2^; O-NO: 68.6 ± 5.4 cm^2^; O-OB: 73.4 ± 16.3 cm^2^, *P* < 0.01, *η*^2^ = 0.36), which were significantly greater in Y-NO compared with O-NO (*P* = 0.001, *d* = 1.86 and *P* = 0.001, *d* = 1.88, respectively) and O-OB (*P* = 0.013, *d* = 1.36 and *P* = 0.043, *d* = 1.01), with no differences between O-NO and O-OB (*P* = 0.878, *d* = 0.08 and *P* = 0.139, *d* = 0.86, respectively, Fig. 3A and B). ITFF (*m.* vastus lateralis, Y-NO: 3.0 ± 1.0%; O-NO: 4.0 ± 0.9%; O-OB: 9.1 ± 2.6%) at 50% quadriceps length was significantly higher in O-OB compared with Y-NO and O-NO (*P* < 0.001, *η*^2^ = 0.75, Fig. 3C), indicating that ITFF was greater in O-NO compared with Y-NO (*P* = 0.030, *d* = 1.07), which was exacerbated by the presence of obesity (*P* < 0.001, *d* = 3.11). Similar observations were found across different quadriceps and/or thigh muscles (Fig. 3C, Supplementary Table 3). Representative cross-sectional images at 50% thigh length for each group are presented in Fig. 3D.

### Quadriceps strength and voluntary activation

**Fig. 4:**
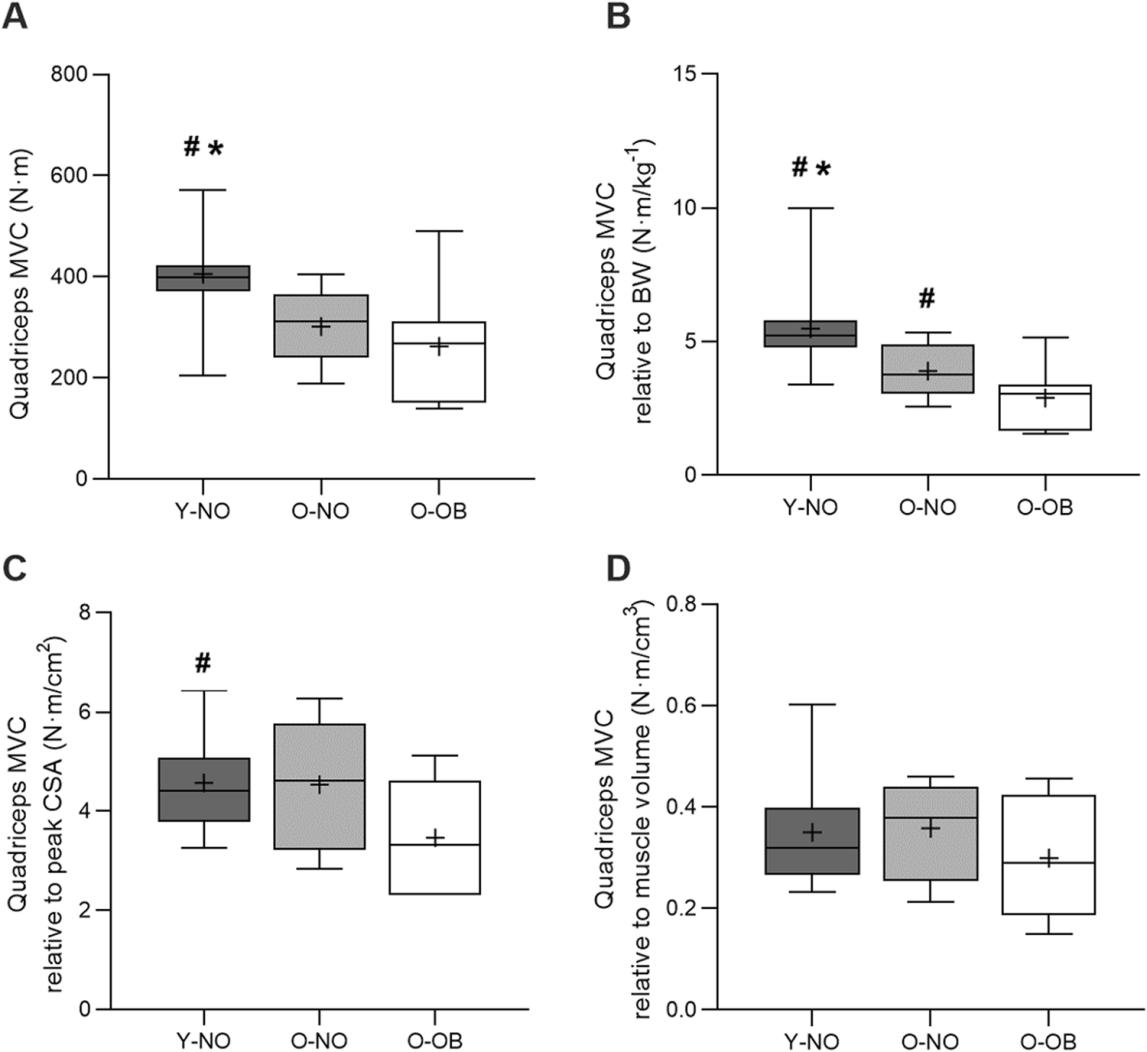
Normalised quadriceps maximal voluntary contraction force (MVC, **A**), quadriceps MVC relative to body mass (BW, **B**), quadriceps MVC relative to quadriceps peak cross-sectional area (CSA, **C**), and quadriceps MVC relative to quadriceps muscle mass (**D**) for young non-obese (Y-NO, dark grey boxes), old non-obese (O-NO, light grey boxes), and old obese (O-OB, white boxes). Values are presented as the median (central horizontal line), 25th and 75th percentiles (box), minimum and maximum values (vertical lines), and mean (cross). Significance was set at *P* < 0.05. * Significantly different from O-NO; # significantly different from O-OB

Quadriceps MVC normalised to patella tendon moment arm length (Y-NO: 406 ± 95 n·m; O-NO: 302 ± 76 n·m; O-OB: 263 ± 106 n·m, *P* < 0.01, *η*^2^ = 0.33, Fig. 4A) was significantly higher in Y-NO compared with O-NO (*P* = 0.008, *d* = 1.21) and O-OB (*P* = 0.002, *d* = 1.42), with no differences between O-NO and O-OB (*P* = 0.355, *d* = 0.42). MVC expressed relative to total body mass (Y-NO: 5.5 ± 1.6 n·m/kg^−1^; O-NO: 3.9 ± 1.0 n·m/kg^−1^; O-OB: 2.9 ± 1.1 n·m/kg^−1^, *P* < 0.0001, *η*^2^ = 0.46, Fig. 4B) was significantly higher in Y-NO than O-NO (*P* = 0.007, *d* = 1.25) and O-OB (*P* = 0.002, *d* = 1.42), and significantly lower in O-OB than O-NO (*P* = 0.047, *d* = 0.95). When MVC was expressed relative to quadriceps muscle peak CSA (Y-NO: 4.6 ± 0.9 n·m/cm^2^; O-NO: O-NO: 4.5 ± 1.3 n·m/cm^2^; O-OB: 3.5 ± 1.2 n·m/cm^2^, *η*^2^ = 0.16, Fig. 4C), Y-NO was significantly higher than O-OB (*P* = 0.037, *d* = 0.101), with no differences between O-NO and Y-NO (*P* = 0.941, *d* = 0.03) and O-OB (*P* = 0.139, *d* = 0.86). However, when MVC was expressed relative to quadriceps muscle volume, there were no significant differences between groups (Y-NO: 0.35 ± 0.10 n·m/cm^3^; O-NO: 0.34 ± 0.09 n·m/cm^3^; O-OB: 0.30 ± 0.13 n·m/cm^3^, *P* = 0.395, *η*^2^ = 0.06, Fig. 4D). There were no differences in quadriceps voluntary activation between groups (Y-NO: 91.1 ± 4.8%; O-NO: 91.8 ± 6.0%; O-OB: 90.1 ± 4.3%, *P* = 0.734, *η*^2^ = 0.02).

### iMyoPS, muscle “activation” and intramuscular signalling

**Fig. 5:**
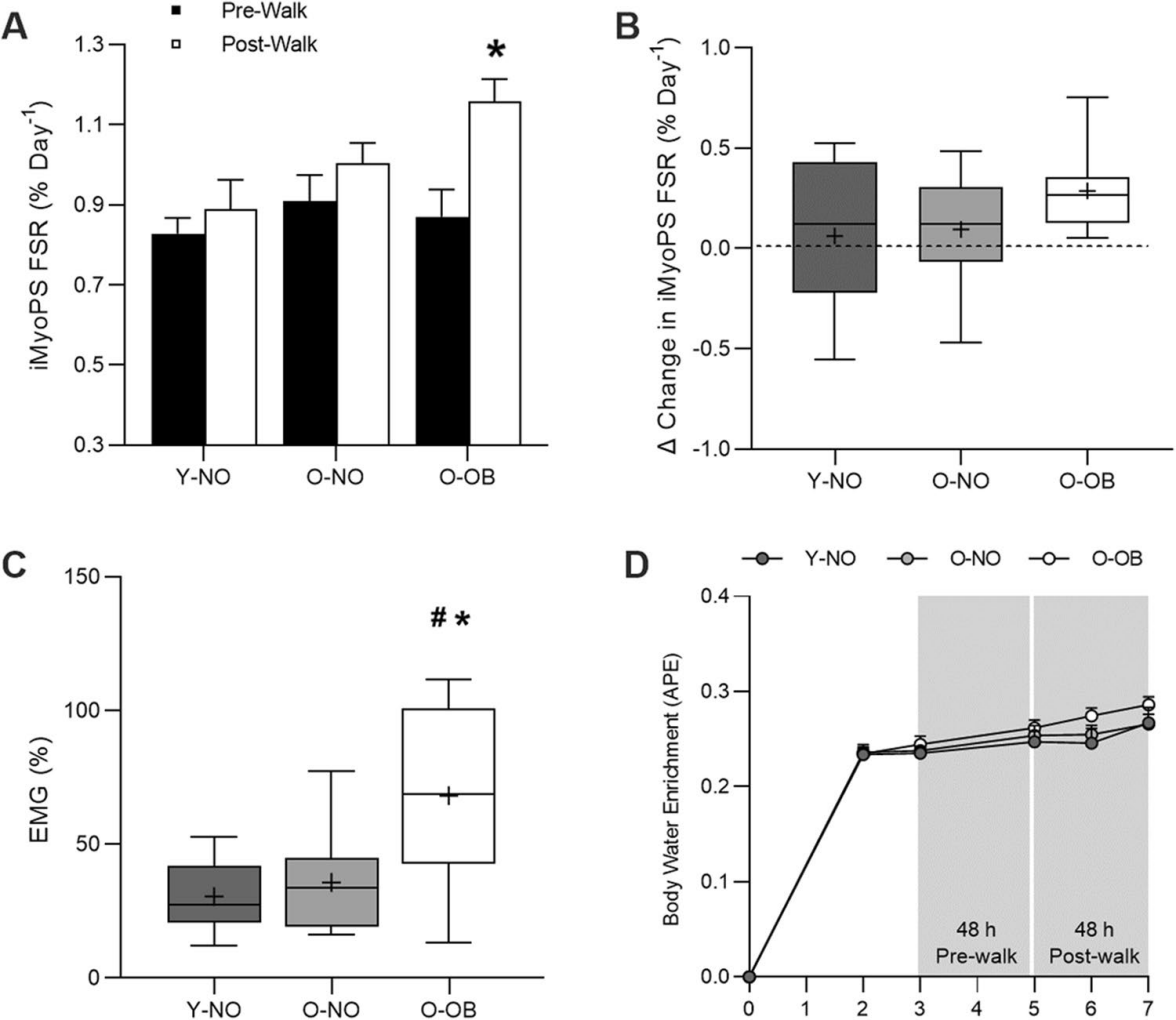
Integrated myofibrillar protein synthesis (iMyoPS) fractional synthetic rates (FSR) over 48 h in the habitual state (black bars) and following a 45-min treadmill walk at ~ 55% V̇O_2peak_ (white bars) in young non-obese (Y-NO), old non-obese (O-ON), and old obese (O-OB), respectively (**A**). Values are presented as means ± SEM. Individual responses are shown by grey circles and grey squares for pre- and post-walk values, respectively. Delta change in integrated myofibrillar protein fractional synthesis rates (FSR) from habitual to 48 h- post-exercise is presented in **B**. Average electromyography (EMG) amplitude, a marker of thigh muscle activity, during the 45-min treadmill walk is presented in **C** and expressed as a percentage of peak EMG amplitude from the progressive walk-to-run test at visit 2. Values are presented as the median (central horizontal line), 25th and 75th percentiles (box), minimum and maximum values (vertical lines), and mean (cross). The dashed horizontal line in **B** represents zero change. Body water deuterium (^2^H) enrichment (atom percent excess; APE) for young non-obese (Y-NO; dark grey circles), old non-obese (O-NO; light grey circles), and old obese (O-OB; clear circles) are presented in **D**. Values are presented as means ± SEM. Significance was set at *P* < 0.05. For **A**, * indicates significant change from pre-post walk in O-OB. For **C**, * significantly different from Y-NO; # significantly different from O-NO

No differences in habitual iMyoPS rates were observed between groups (Y-NO: 0.83 ± 0.04%·day^−1^; O-NO: 0.91 ± 0.07%·day^−1^; O-OB: 0.87 ± 0.07%·day^−1^, *P* = 0.564, *η*^2^ = 0.04, Fig. 5A). A mixed model ANOVA revealed significant main effects for time (*P* = 0.007, *η*^2^ = 0.21) and group (*P* = 0.043,* η*^*2*^ = 0.18) on iMyoPS; however, no interaction effect (i.e. time × group) was observed (*P* = 0.189, *η*^2^ = 0.10). However, while we observed a significant main effect of time in the absence of an interaction effect, the 48-h delta change in weight-bearing-induced iMyoPS rates was statistically significant in O-OB only (+ 39 ± 12% delta change, *P* = 0.005, *d* = 1.54) and not Y-NO and O-NO (Y-NO: 0.06 ± 0.09%·day^−1^; O-NO: 0.10 ± 0.09%·day^−1^; O-OB: 0.29 ± 0.07%·day^−1^, both *P* > 0.271, Fig. 5A, 5B). Average EMG amplitude during the 45-min. treadmill walk, expressed as a percentage of the maximal EMG amplitude recorded during the progressive walk-to-run test (Y-NO: 30.5 ± 13.5%; O-NO: 35.8 ± 19.7%; O-OB: 68.3 ± 32.3%, *P* < 0.01, *η*^2^ = 0.38, Fig. 5C), was significantly higher in O-OB than O-NO (*P* = 0.027, *d* = 1.21) and Y-NO (*P* = 0.001, *d* = 1.53). Body water ^2^H enrichments are presented in Fig. 5D. Briefly, body water ^2^H enrichment, assessed via saliva samples, increased significantly above rested-state values at 24 h after consumption of the first dose in all groups (Y-NO: 0.23 ± 0.02 APE, O-NO: 0.24 ± 0.03 APE; O-OB: 0.24 ± 0.03 APE, all *P* < 0.0001, Fig. 5D). Steady-state isotopic enrichment was maintained throughout the remainder of the study, with no significant difference between groups at any time point (all *P* > 0.97, Fig. 5D). No differences in intramuscular anabolic signalling were observed at any time point within or between groups (all *P* > 0.05, Supplemental Fig. 1).

## Discussion

Age-related muscle loss is typically accompanied by a concomitant increase in whole-body adiposity and ectopic fat deposition within skeletal muscle [3, 23, 25]. Skeletal muscle anabolic resistance to amino acids, which contributes to age-related muscle loss, is exacerbated by obesity through an impaired metabolic phenotype, accumulation of intramyocellular lipids (myosteatosis), dyslipidaemia, and adipose tissue–derived inflammation [5, 13, 23, 50, 56, 61, 63]. In addition, obesity is also typically associated with sedentarism, reduced physical activity, and poor diet quality [68], factors which accelerate age-related muscle deterioration. Despite this, absolute levels of lean mass have been shown to be equivalent or greater in older obese compared with older non-obese individuals [50, 61]. Conversely, the age-related decline in muscle quality appears to be aggravated by the presence of obesity [22, 32, 35, 52, 59]. We hypothesised that equivalent or greater absolute muscle mass in older obese (i.e. O-OB) compared with non-obese older (i.e. O-NO) and younger (i.e. Y-NO) individuals might be due to a greater muscle anabolic response to weight-bearing physical activity, through increased loading forces and muscle contractile work during activities of daily living. We also postulated that age-related indices of muscle quality would be worsened in O-OB compared with O-NO and Y-NO. In agreement with our hypotheses, in response to a weight-bearing loading task designed to mimic a typical brisk walk, iMyoPS increased above habitual values in O-OB, but not O-NO and Y-NO. Despite equivalent quadriceps muscle mass between O-OB and O-NO (that was lower than Y-NO in both older groups), indices of muscle functional and metabolic quality (e.g. relative strength and intramuscular fat) were worsened in O-OB compared with O-NO.

The present data demonstrate that rates of integrated myofibrillar protein synthesis (iMyoPS) were increased above habitual values over 48-h after a 45-min weight-bearing walking task in O-OB only (39%), but not Y-NO and O-NO (11% and 12%, respectively). Although we may have been underpowered to detect a significant interaction effect in the iMyoPS response to the walking task, the large effect size for both absolute and delta change in iMyoPS for O-OB only indicates significantly elevated rates in this cohort. The iMyoPS response to weight-bearing activity in O-OB was likely due to the mechanical stimulus of a greater loading force (i.e. moving more inert mass and a greater absolute body mass) as previously postulated [28, 48]. Indeed, quadriceps muscle “activity’, assessed using average surface EMG amplitude across the 45-min task, was significantly greater in O-OB compared with Y-NO and O-NO (68% of maximal EMG amplitude compared with 31% and 36% in Y-NO and O-NO, respectively). These data are consistent with previous observations suggesting an increase in muscle contractile work for obese individuals when walking and greater load carriage of weight-bearing muscles (e.g. [54]. That diet and physical activity were kept constant during iMyoPS assessment also suggests that the increase in iMyoPS in O-OB was a direct response to the weight-bearing walking task. It is also worth noting that, although daily step count was greater than habitual values on the day of the 45-min walk in O-OB and O-NO, iMyoPS increased above habitual rates in O-OB only. The discrepancy between the current iMyoPS data and previous findings of a similar or impaired muscle anabolic response to muscle mechanical contraction in young and middle-aged obese individuals [4, 26, 30] is likely due to differences in muscle contractile loading between seated resistance or aerobic exercise and weight-bearing activity. Nonetheless, it appears that obesity impairs muscle adaptive remodelling to traditional exercise training modalities, independently of ageing. Given that O-OB in this study were relatively active (~ 8500 daily steps on average) and lower-risk obese (~ 31 kg·m^−2^, 33% body fat), we cannot rule out that the iMyoPS response to weight-bearing activity is diminished in more sedentary and/or severely O-OB individuals. The physical activity status of O-OB also supports the notion that the increase in iMyoPS following weight-bearing activity is not a response to damage, degradation, and/or synthesis imbalances through exposure to an unaccustomed task (K. E. [7, 18, 57].

Previously, we have demonstrated that the acute myofibrillar MPS response to moderate-dose protein ingestion at rest was diminished in O-OB compared with Y-NO and O-NO, and was associated with leg fat mass and physical activity levels [61]. Resistance exercise contraction (e.g. [20] and moderate-intensity treadmill walking (e.g. [65] have been shown to augment postprandial muscle anabolism in young and older adults (i.e. alleviate anabolic resistance) by increasing the use of dietary-derived amino acids for MPS (e.g. [55]. Furthermore, resistance exercise and load-carriage can stimulate an increase in postabsorptive MPS (e.g. [54]. Hence, it is possible that the observed increase in iMyoPS rates in O-OB over 48 h after weight-bearing physical activity may be underscored by augmented postabsorptive and/or postprandial MPS, although our study design did not enable us to determine this. Notwithstanding, the measure of iMyoPS using D_2_O ingestion is a less invasive alternative to acutely measured MPS protocols, which determines muscle anabolism under “free-living” conditions, incorporating all feeding and physical activity events, and is associated with longer-term muscle mass change in older adults [10, 11].

Interestingly, habitual iMyoPS rates over 48 h prior to the walking task did not differ between groups. Given earlier evidence of impaired postprandial MPS in O-NO and O-OB compared with Y-NO [5, 50, 61], and the notion that cumulative deficits in MPS drive the progression of sarcopenia [10, 11], it would be reasonable to expect habitual iMyoPS to be lower in O-OB and O-NO compared with Y-NO. It is possible that the diet and activity control measures implemented during the assessment of iMyoPS and/or the relatively short 48-h timeframe of assessment could have impacted our ability to detect subtle between-group differences in iMyoPS. We also speculate that the degree of weight-bearing activity in O-OB over the 48-h habitual phase may have provided sufficient stimulus to counteract any postprandial muscle anabolic resistance, thereby eliciting habitual iMyoPS rates that were indistinguishable from O-NO. Nonetheless, acute postabsorptive and postprandial myofibrillar fractional synthesis rates on the order of 0.02–0.06%·h^−1^ have been widely reported in young and older individuals (e.g. [46, 61], suggesting the present habitual “free-living” iMyoPS rates of 0.6–1.4%·day^−1^ are within the expected physiological range. Given the potential importance of altered muscle protein breakdown with ageing and obesity [17, 50, 66], this locus of muscle mass regulation warrants further investigation. Similarly, our analysis of rested and immediate post-walk intramuscular signalling regulators of MPS revealed no differences within or between groups, suggesting that more detailed temporal signalling work may be required to understand the molecular regulators of muscle mass in O-OB.

An increased iMyoPS response to weight-bearing activity in O-OB, in the face of any postprandial muscle anabolic resistance (i.e. in response to amino acid nutrition), may provide the mechanistic basis for the equivalent absolute quadriceps muscle volume/CSA between O-OB and O-NO. That absolute muscle volume and CSA was not greater in O-OB compared with O-NO may be due to the low-risk obesity status in this cohort or variability in our relatively small sample. Indeed, others have shown greater muscle mass (and markers of) in more severely obese older adults (e.g. [70–72]. Although not determined herein, it is likely that muscle fibre CSA may have been greater in O-OB compared with O-NO, as we have previously demonstrated [61]. Importantly, the brisk walk task in the current study increased step-count well above habitual levels in O-OB. Therefore, it is unlikely that O-OB would regularly stimulate iMyoPS rates to the same robust levels observed over 48-h post-walk task that would theoretically promote greater muscle mass in O-OB over O-NO. Furthermore, relative daily protein intake for O-OB (0.94 g⋅ kg^−1^) was also below recommendations for longer-term muscle retention in older adults [51], which would likely be deleterious to muscle mass accretion, particularly in the face of postprandial muscle anabolic resistance in O-OB. Knowledge of whether muscle mass in non-load bearing regions is equivalent or greater in O-OB over O-NO would provide clarity to our hypothesis that weight-bearing activity induces muscle anabolism and supports absolute muscle mass in O-OB. Any such future work would also have important implications for other basic physiological functions (e.g. breathing, lifting, mobilising, eating) that may decline rapidly in the presence of the obese metabolic phenotype [14, 60]. While our data provide a valuable contribution to our understanding of the relationship between obesity, ageing, and muscle regulation, these data should be interpreted cautiously as it is not known how persistent this effect may be in older obese men and/or if they are present in women. Indeed, the effects of obesity on muscle protein turnover in pre-sarcopenic middle-aged, severely obese as well as frail (i.e. exhibiting very poor function), and oldest-old males and females need to be comprehensively assessed.

Muscle quality, defined as strength and/or power per unit of muscle mass, is lost more rapidly than absolute muscle mass with advancing age [24, 42] and represents a significant contributor to the impairment in physical function [42, 64], particularly in obese individuals with a larger body mass to carry. Indeed, it is generally accepted that obese individuals, independent of age, have lower maximal strength when expressed relative to body mass (e.g. [39, 50]. In the present study, no differences in absolute MVC were observed between O-NO and O-OB, though age-related impairments were found. However, MVC relative to total body mass was lower in O-NO compared with Y-NO and was exacerbated further in O-OB, consistent with previous findings (e.g. [50, 62, 69–71],Tomlinson, Erskine, Winwood, et al. 2014a). The difference between O-NO and O-OB in MVC relative to peak quadriceps volume/CSA was not statistically significant, which may be due to the relatively low sample size and large variability in relative MVC measures. Nonetheless, a large effect size (*d* = 0.86) in MVC/peak CSA was apparent between O-OB and O-NO. Indeed, previous studies demonstrate that the age-related decline in muscle quality, assessed via markers of in-situ muscle-specific force, is exacerbated by obesity, even in the presence of increased strength and muscle volume [67],Tomlinson, Erskine, Winwood, et al. 2014a, 2014b; [72]. This is important as reduced relative lower-limb strength can manifest with impaired functional capacity (e.g. difficulty walking, climbing stairs, rising from a chair/bed) [38], and increased risk of joint pathologies [15], that would have a profound impact on overall quality of life in O-OB. Interestingly, quadriceps voluntary activation did not differ between groups, which contrasts with previous studies suggesting that obesity is associated with impaired voluntary recruitment of muscle (e.g. [8, 9, 40, 53]. The relatively low-risk obese and high functional status of O-OB in the present study may have impacted our ability to detect a clear deficit in voluntary activation. Indeed, in more advanced metabolic disease and severe O-OB individuals, declines in muscle functional quality and voluntary activation appear to be more pronounced (J. A. [6, 19, 68],Tomlinson, Erskine, Winwood, et al. 2014a, 2014b). Collectively, our findings provide some evidence of lower muscle functional quality with ageing that may be exacerbated by the presence of obesity. The driving mechanisms of lowered muscle quality in O-OB may be partly modulated by lipid-induced alterations, including systemic inflammation, intracellular lipotoxicity, impaired muscle structure/contractility, or impaired skeletal muscle regenerative capacity (*for review* [48], which warrants further exploration.

Infiltration of fat within skeletal muscle is apparent with ageing and exacerbated by the presence of obesity via lipid overspill, with implications for metabolic health and muscle structure/contractility [3, 22, 23, 25, 62, 63]. In the present study, quadriceps ITFF, a marker of fat infiltration within the muscle, was significantly elevated in O-NO compared with Y-NO and even greater in O-OB, which is consistent with previous findings [21, 41, 45]. Although we did not directly measure intramyocellular lipid content at a microscopic level, we previously reported a robust two-fold greater type II fibre lipid infiltration O-OB compared with O-NO that coincided with greater postprandial muscle anabolic resistance [61]. Intuitively, the greater ITFF in O-OB compared with O-NO would partly explain their worsened metabolic phenotype (markers of insulin resistance and systemic inflammation) and signs of poorer muscle functional quality. Considering the metabolic and functional health status of our O-OB cohort relative to more severe O-OB cohorts included in previous studies (e.g. [70–72], it is possible that differences in muscle functional quality would diverge further from O-NO as metabolic disease progresses to an advanced stage [2, 68–71]. Whether the abundance, species, or sub-cellular location of intramuscular lipids influences indices of muscle quality in O-OB has yet to be explored. Collectively, the equivalent or greater absolute muscle mass in O-OB over O-NO is concurrent with greater intramuscular fat, which has dire consequences for muscle function, metabolic health, and overall quality of life.

In conclusion, we demonstrate here that weight-bearing activity stimulated an increase in 48-h iMyoPS rates above habitual values in O-OB, but not O-NO and Y-NO. These data may explain observations of equivalent or greater muscle mass in O-OB compared with O-NO and Y-NO, with weight-bearing activity potentially counteracting acute postprandial muscle anabolic resistance previously shown in O-OB. In line with the worsened metabolic phenotype and greater ITFF of O-OB, the present data suggests that the age-related decline in indices of muscle quality may be aggravated by the presence of obesity, the mechanistic basis of which requires further attention. Finally, the effects of obesity on muscle protein turnover in pre-sarcopenic middle-aged, severely obese, as well as frail and oldest-old males and females need to be comprehensively assessed to develop feasible and effective interventions for fat mass reduction and muscle mass maintenance/growth that will combat rising sarcopenic-obesity prevalence.

### Supplementary Information

Below is the link to the electronic supplementary material.

Fig. S1 Intramuscular signaling of (A) 4E-BP1Thr37/46, (B) AMPKThr72, (C) AktSerine 473, (D) rpS6Ser235/236, (E) IRS-1Ser636/639 and (F) TSC2Thr1462 at pre- and post-walk in young non-obese (Y-NO, n=14), older non-obese (O-NO, n=8) and older obese (O-OB, n=6), respectively. All proteins are expressed relative to their respective total protein abundance. Muscle samples were obtained in a fasted state, pre- (black bars) and immediately post- (white bars) walk (visit 5). Individual responses are shown by grey circles and grey squares for pre- and post-walk values, respectively. Significance was set at P<0.05. Values are presented as means ± SD and expressed as fold change from Y-NO pre-walk. Representative blot images are shown on the top of each respective panel for Y-NO, O-NO and O-OB (from left-to-right) pre- (R) and post-walk (W), respectively.(PNG 624 kb)

High Resolution (TIF 330 kb)

Supplementary file2 (DOCX 13 KB)

Supplementary file3 (DOCX 18 KB)

## Data Availability

The raw data supporting the conclusions of this article will be made available by the authors, without undue reservation.
