# Supplementary figures and images for: Greater myofibrillar protein synthesis following weight-bearing activity in obese old compared with non-obese old and young individuals
Source: GeroScience. 2023 Jun 17;46(4):3759–78. doi: 10.1007/s11357-023-00833-2 (PMC11226697; doi:10.1007/s11357-023-00833-2)

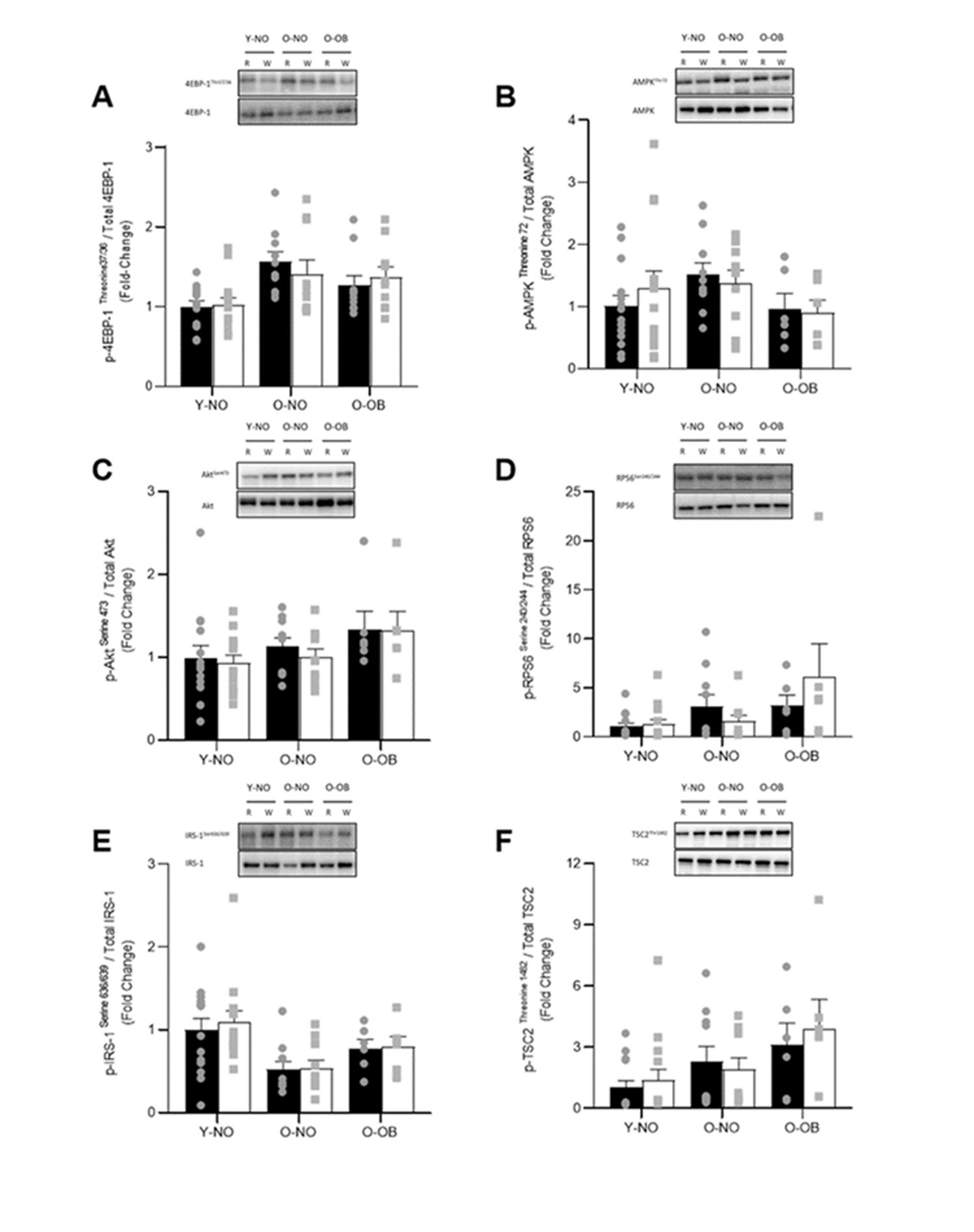

Supplement: Supplementary file 1 — Intramuscular signaling of (A) 4E-BP1Thr37/46, (B) AMPKThr72, (C) AktSerine 473, (D) rpS6Ser235/236, (E) IRS-1Ser636/639 and (F) TSC2Thr1462 at pre- and post-walk in young non-obese (Y-NO, n=14), older non-obese (O-NO, n=8) and older obese (O-OB, n=6), respectively. All proteins are expressed relative to their respective total protein abundance. Muscle samples were obtained in a fasted state, pre- (black bars) and immediately post- (white bars) walk (visit 5). Individual responses are shown by grey circles and grey squares for pre- and post-walk values, respectively. Significance was set at P<0.05. Values are presented as means ± SD and expressed as fold change from Y-NO pre-walk. Representative blot images are shown on the top of each respective panel for Y-NO, O-NO and O-OB (from left-to-right) pre- (R) and post-walk (W), respectively.(PNG 624 kb) [file 11357_2023_833_Fig6_ESM.png]

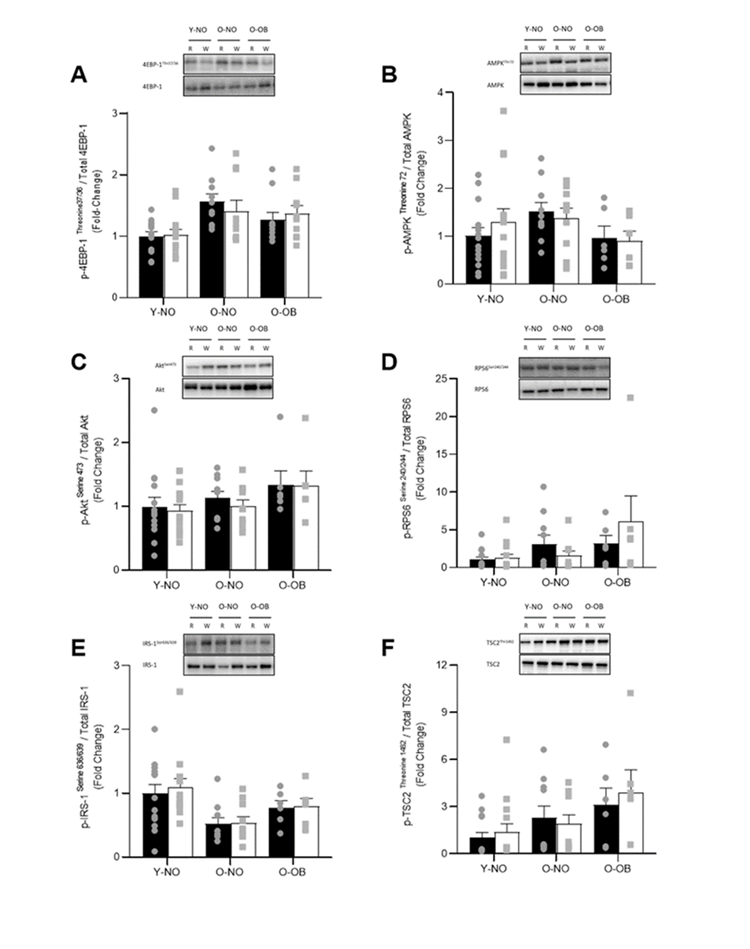

Supplement: Supplementary file 2 — High Resolution (TIF 330 kb) [file 11357_2023_833_MOESM1_ESM.tif]
