# Supplementary material for: Greater myofibrillar protein synthesis following weight-bearing activity in obese old compared with non-obese old and young individuals
Source: GeroScience. 2023 Jun 17;46(4):3759–78. doi: 10.1007/s11357-023-00833-2 (PMC11226697; doi:10.1007/s11357-023-00833-2)
Supplement: Supplementary file 3 — Supplementary file2 (DOCX 13 KB) [file 11357_2023_833_MOESM2_ESM.docx]

******************************* Supplementary Methods *******************************

**Supplementary methods**

*Primary antibodies for immunoblotting*

Membranes were blocked in 5% low‐fat milk (diluted in Tris‐buffered saline and 0.1% Tween‐20 [TBS‐T]) for 1 h at room temperature and then incubated in a rocking device overnight at 4°C in the presence of the following primary antibodies (1:1000 dilution in 2.5% BSA in TBS‐T): p-IRS-1^Ser636/639^ (CST, 2388, 1:1000), IRS-1 (CST, 2382, 1:1000), p-Akt^Ser473^ (CST, 4051, 1:1000), Akt (CST, 4691, 1:1000), p-TSC2^Thr1462^ (CST, 3617, 1:1000), TSC2 (CST, 4308, 1:1000), AMPK^Thr72^ (CST, 2535, 1:1000), AMPK (CST, 2532, 1:1000), p-RPS6^Ser240/244^ (CST, 2215, 1:1000), RPS6 (CST, 2217, 1:1000). For each, membranes were washed 3 × 5 min in TBS‐T, incubated in HRP-linked anti-rabbit (CST7074, 1:10000 in TBST) or anti-mouse (CST7076, 1:10000 TBST) IgG at room temperature for 1 h, before 3 × 5 min washes in TBS‐T. Finally, membranes were exposed to chemiluminescent HRP Substrate (Millipore Corp., Billerica, MA, USA) for 2-3 minutes and visualized using a BOX Chemi XT4 Imager with GeneSys capture software (Syngene, Cambridge, UK). Bands were quantified using ImageJ software (National Institute of Health, Bethesda, MD). Relative arbitrary units were normalized to the total amount of protein loaded as visualized via Ponceau S staining. Where appropriate, the phosphorylation of proteins as a proxy of their activation is expressed relative to the total abundance of protein.

******************************* Supplementary Methods *******************************
