# Supplementary material for: Greater myofibrillar protein synthesis following weight-bearing activity in obese old compared with non-obese old and young individuals
Source: GeroScience. 2023 Jun 17;46(4):3759–78. doi: 10.1007/s11357-023-00833-2 (PMC11226697; doi:10.1007/s11357-023-00833-2)
Supplement: Supplementary file 4 — Supplementary file3 (DOCX 18 KB) [file 11357_2023_833_MOESM3_ESM.docx]

******************************** Supplementary Table 1 ********************************

Supplemental Table 1. Modified Bruce protocol

| **Stage** | **Speed (km/hr)** | **Speed (mph)** | **Gradient** |
| --- | --- | --- | --- |
| **1** | 2.74 | 1.7 | 0 |
| **2** | 2.74 | 1.7 | 5 |
| **3** | 2.74 | 1.7 | 10 |
| **4** | 4.02 | 2.5 | 12 |
| **5** | 5.47 | 3.4 | 14 |
| **6** | 6.76 | 4.2 | 16 |
| **7** | 8.05 | 5 | 18 |
| **8** | 8.85 | 5.5 | 20 |
| **9** | 9.65 | 6 | 22 |
| **10** | 10.46 | 6.5 | 24 |
| **11** | 11.26 | 7 | 26 |
| **12** | 12.07 | 7.5 | 28 |
|  |  |  |  |
| Modified Bruce protocol with 2-min stages | | | |

******************************** Supplementary Table 1 ********************************

******************************** Supplementary Table 2 ********************************

Supplemental Table 2. Prescribed dietary characteristics

|  | **Y-NO** | **O-NO** | **O-OB** |  |  |  |
| --- | --- | --- | --- | --- | --- | --- |
| **Daily energy intake** (kcal) | 2,816 ± 246 | 2,393 ± 138 * | 2,600 ± 220 * ^#^ |  |  |  |
| **Daily protein** (g⋅kg^−1^) | 1.42 ± 0.0 | 1.16 ± 0.03 * | 1.10 ± 0.02 * ^#^ |  |  |  |
| **Daily CHO** (g⋅kg^−1^) | 5.25 ± 0.25 | 4.25 ± 0.12 * | 3.96 ± 0.07 * ^#^ |  |  |  |
| **Daily fat** (g⋅kg^−1^) | 1.27 ± 0.06 | 1.03 ± 0.03 * | 0.96 ± 0.02 * ^#^ |  |  |  |
|  |  |  |  |  | |  |
| Data presented as mean ± standard deviation. * Significantly different from Y-NO, ^#^ significantly different from O-NO. The wight maintenance diet was prescribed at ∼50% carbohydrate, ∼30% fat and ∼20% protein. Abbreviations: Y-NO, young non-obese; O-NO, older-non-obese; O-OB, older obese; CHO, carbohydrate. | | | | |  |  |

******************************** Supplementary Table 2 ********************************

******************************** Supplementary Table 3 ********************************

Supplemental Table 3. Total (whole) Thigh Muscle Characteristics

|  | **Y-NO** | **O-NO** | **O-OB** |  |
| --- | --- | --- | --- | --- |
| **Patella tendon moment arm length** (mm) | 35.6 ± 2.5 | 36.1 ± 1.4 | 35.0 ± 1.6 |  |
| **ITFF** |  |  |  |  |
| ***m.Vastus lateralis*** *(%)* | 3.0 ± 1.0 | 4.0 ± 1.0 * | 9.0 ± 2.8 * ^#^ |  |
| ***m.Vastus medialis*** *(%)* | 2.4 ± 0.4 | 3.9 ± 0.6 * | 5.5 ± 2.5 * ^#^ |  |
| ***m.Rectus femoris*** *(%)* | 2.0 ± 0.7 | 2.2 ± 1.2 | 3.7 ± 0.9 * ^#^ |  |
| ***Total quadriceps muscle*** *(%)* | 2.0 ± 0.5 | 3.8 ± 1.1 * | 7.0 ± 1.5 * ^#^ |  |
| **Total thigh fat fraction** (%) | 26.6 ± 9.4 | 32.3 ± 4.7 * | 42.4 ± 6.3 * ^#^ |  |
| **Total thigh muscle volume** (cm^3^) | 1,845 ± 358 | 1.663 ± 232 * | 1,790 ± 505 * ^#^ |  |
| **Peak CSA** | 89.0 ± 14.4 | 68.6 ± 5.4 * | 73.4 ± 16.3 * |  |
| **Mean quadriceps CSA** (cm^2^) | 76.9 ± 12.0 | 61.6 ± 6.3 * | 62.6 ± 17.2 * |  |
|  |  |  |  |  |
| Data presented as mean ± standard deviation. Total thigh fat fraction includes subcutaneous fat. Mean quadriceps CSA taken from 50% thigh portion. * Significantly different from Y-NO, ^#^ significantly different from O-NO. Abbreviations: Y-NO, young non-obese; O-NO, older-non-obese; O-OB, older obese; ITFF, intramuscular thigh fat fraction; CSA, cross sectional area (of quadriceps); *m*, muscle. | | | | |

******************************** Supplementary Table 3 ********************************
